# Effect of glycaemic control on growth factor release and *in-vitro* biological properties of advanced and injectable platelet-rich fibrin in type 2 diabetes Mellitus

**DOI:** 10.3389/fdmed.2026.1895392

**Published:** 2026-08-11

**Authors:** Rajesh Kashyap Shanker, Anupama Rao, Ashwini Prabhu, Vijaya Kumar, Sowmiya Narayani, Ranajit Das, Aparna Hegde

**Affiliations:** 1 Department of Periodontology, Yenepoya Dental College, Yenepoya (Deemed to be University), Mangaluru, Karnataka, India; 2 Yenepoya Research Centre, Yenepoya (Deemed to be University), Mangaluru, Karnataka, India

**Keywords:** A-PRF, growth factors, i-PRF, periodontal regeneration, platelet-rich fibrin, type 2 diabetes mellitus

## Abstract

**Background:**

Platelet-rich fibrin has shown regenerative potential in periodontal treatments; nonetheless, patients with Type 2 diabetes mellitus (T2DM) may experience altered biological activities. Therefore, this study aimed to evaluate the regenerative potential and growth factor release profiles of advanced platelet-rich fibrin (A PRF) and injectable platelet-rich fibrin (i-PRF) derived from participants with controlled and uncontrolled T2DM.

**Methods:**

This study compared *n* = 87 (29 per group) participants—controlled T2DM, uncontrolled T2DM, and Healthy non-diabetic groups—analysing blood samples for HbA1c, platelet count, and growth factors like Vascular endothelial growth factor (VEGF), Platelet-derived growth factor-BB (PDGF-BB), Insulin-like growth factor-1 (IGF-1), Transforming growth factor-beta 1 (TGF-*β*1), Transforming growth factor-beta 2 (TGF-*β*2) via Enzyme-linked immunosorbent assay (ELISA), assessing cytocompatibility with 3-(4,5-dimethylthiazol-2-yl)-2,5-diphenyltetrazolium bromide (MTT) assays, and employing statistical methods like Analysis of variance (ANOVA) correlation, and regression.

**Results:**

PRF derived from participants with uncontrolled T2DM demonstrated significantly reduced PDGF-BB and TGF-*β*2 release compared with healthy participants in both A-PRF (*P* = 0.016), i-PRF (*P* = 0.033), and A-PRF (*P* = 0.002) and i-PRF (*P* = 0.0034. VEGF release was significantly lower in uncontrolled T2DM than in well-controlled T2DM in both A-PRF (*P* = 0.0096) and i-PRF (*P* = 0.0023). Compared with the uncontrolled T2DM group, well-controlled T2DM exhibited significantly higher VEGF release in both A-PRF (*P* = 0.0096) and i-PRF (*P* = 0.0023), and significantly higher IGF-1 (*P* < 0.05) and TGF-*β*2 (*P* = 0.033) levels in i-PRF. Platelet counts were comparable across the study groups, whereas cell viability was highest in PRF from healthy participants. HbA1c was not independently associated with the evaluated growth factor concentrations in multivariable analyses.

**Conclusions:**

A-PRF and i-PRF derived from participants with well-controlled T2DM exhibited biological properties comparable to those of healthy non-diabetic individuals, whereas uncontrolled T2DM was associated with reduced release of key growth factors, indicating compromised healing potential. These findings suggest that achieving good glycaemic control may optimize the biological performance of PRF for periodontal regenerative therapy. However, the present findings are based on *in vitro* analyses; confirmation through well-designed prospective clinical studies remains essential before these findings can be translated into routine clinical practice.

## 1 Introduction

Periodontitis is a highly prevalent chronic inflammatory oral disease that damages the tooth-supporting structures, affecting approximately 20%–50% of the global population (1) and accounting for an estimated 1.1 billion cases in 2019, with a 68% increase in the prevalence of severe periodontitis since 1990 (2). If left untreated, it results in progressive destruction of the periodontal ligament and alveolar bone, ultimately leading to tooth loss in nearly 276 million individuals worldwide (3). Although dental plaque is the primary etiological factor, the onset, severity, and progression of periodontitis are strongly influenced by systemic conditions, particularly diabetes mellitus (DM). India has the second-largest population of individuals with diabetes globally, with an estimated 77 million affected adults (4). Individuals with type 2 diabetes mellitus (T2DM) have a two- to three-fold greater risk of developing periodontitis (5), and this increased susceptibility is closely related to the degree of glycaemic control (6).

In this context, platelet-rich fibrin (PRF), first introduced by Choukroun et al. (7), has emerged as a second-generation autologous platelet concentrate prepared without anticoagulants and shows promising regenerative potential in periodontal therapy. PRF has been investigated in a wide range of clinical applications, including periodontal regeneration, implant dentistry, extraction socket preservation, sinus augmentation, management of gingival recession, and oral and maxillofacial surgery (8).

PRF contains several growth factors that enhance wound healing, angiogenesis, bone regeneration, graft stabilization, and hemostasis. Based on the low-speed centrifugation concept, Advanced PRF (A PRF) and injectable PRF (i-PRF) have gained particular interest due to their improved biological characteristics, owing to their greater cellular and growth factor content and sustained release. A PRF has been demonstrated to stimulate the release of key growth factors involved in wound healing, thereby exerting a beneficial effect on human gingival fibroblasts (9). Several randomized clinical trials and subsequent systematic reviews have reported improvements in probing pocket depth reduction, clinical attachment level gain, and radiographic bone fill when PRF is used as an adjunct to periodontal regenerative procedures. However, the magnitude of these benefits has varied across studies due to differences in PRF preparation protocols, centrifugation systems, defect morphology, adjunctive regenerative materials, and study methodologies, resulting in heterogeneous evidence regarding its clinical effectiveness (10, 11). Additionally, a study evaluating platelet-rich plasma(PRP) in diabetic participants with diabetic foot ulcers found no significant difference in growth factor release between diabetic and Healthy non-diabetic groups, suggesting its potential applicability in regenerative procedures among diabetic participants (12).

However, emerging evidence indicates that diabetes may influence the structural integrity and biological performance of PRF. Alterations in fibrin architecture, cellular distribution, tensile strength, and growth factor release patterns have been reported in diabetic participants (13, 14). Hyperglycaemia-induced modifications in fibrinogen structure, impaired angiogenesis, chronic low-grade inflammation, and delayed wound healing may collectively compromise PRF quality (15). Furthermore, hyperglycaemia has been reported to influence PRF clot characteristics and biological properties in relation to glycaemic control levels (16), highlighting the need for targeted evaluation of PRF preparations in diabetic populations.

Despite the increasing clinical application of PRF, there remains limited evidence regarding the biological quality of A-PRF and i-PRF in individuals with T2DM, particularly regarding how glycaemic control influences their biological performance. While previous studies have suggested that diabetes may affect PRF composition, fibrin architecture, and growth factor release, it remains unclear whether well-controlled and uncontrolled T2DM differentially influence the biological properties of A-PRF and i-PRF to a biologically relevant extent. Furthermore, the relationship between platelet count and functional growth factor releases in PRF preparations derived from diabetic individuals remains inadequately understood. Addressing these uncertainties is important, as platelet count alone may not accurately predict the biological activity of PRF under metabolically compromised conditions. Therefore, the primary objective of the present study was to compare the growth factor release profiles of autologous A-PRF and i-PRF obtained from participants with controlled and uncontrolled T2DM, as well as from healthy non-diabetic individuals. The secondary objectives were to evaluate the relationship between platelet count and growth factor release, assess the association between HbA1c levels and PRF-derived growth factors, and determine the cytocompatibility of PRF preparations using a cell viability assay. We hypothesized that PRF derived from participants with well-controlled T2DM would exhibit biological properties comparable to those of healthy non-diabetic participants, whereas PRF obtained from individuals with uncontrolled T2DM would demonstrate reduced release of selected growth factors and altered biological performance.

## 2 Materials and methods

### 2.1 Ethics approval and consent to participate

The study was carried out in the Department of Periodontology, Yenepoya Dental College, Yenepoya (deemed to be University), Mangaluru, India, from December 8, 2022, to November 21, 2023. Before enrollment, all participants provided written informed consent. On March 21, 2022, the Institutional Ethics Committee of Yenepoya (deemed to be University) approved the study protocol (approval no: YEC-1/2022/037).

### 2.2 Sample collection

The present study was an observational cross-sectional study with laboratory-based analyses of PRF preparations, including a nested *in vitro* cell viability assay that employed a convenience sampling technique. Participants were recruited from individuals who reported to the Department of Periodontology, Yenepoya Dental College, for routine dental examinations or periodontal therapy.

Participants aged 35–65 years were enrolled in the study. Controlled T2DM with HbA1c levels between 6% and 7.9%, uncontrolled T2DM participants with HbA1c levels greater than 8% to 10%, and non-diabetic participants with HbA1c levels ranging from 4% to 5.6% were included in the study. Participants who received steroid therapy, smoked, had other systemic diseases, were pregnant or lactating, or received medications known to influence platelet function or wound healing were excluded from the study. The sample size calculation was based on the pooled standard deviation of PRP platelet count ( × 10^3^/µL) in non-diabetic and diabetic participants (12), which was 182.245, with a mean difference of 155, an effect size of 0.8231, alpha error of 5%, and study power of 80% using a two-sided test. Platelet count was considered the primary endpoint for sample size estimation. As no published data on PRF platelet count variability were available at the time of study design, the sample size calculation was based on platelet count data reported in a previously published PRP study (12). Since both PRP and PRF are autologous platelet concentrates prepared from whole blood and share similar biological characteristics, the PRP study was considered an appropriate reference for estimating the sample size (17, 18). The required sample size was determined to be 29 participants per group, for a total of 87 participants. This calculation was performed using nMaster software, version 2, at the Biostatistics Resource and Training Center, CMC Vellore.

A total of 24 mL of blood was collected from three groups: controlled T2DM participants (*n* = 29), uncontrolled T2DM participants (*n* = 29), and Healthy non-diabetic participants (*n* = 29). Of the total volume, 2 mL was allocated for HbA1c estimation, 2 mL for platelet count analysis, 10 mL for the preparation of A PRF, and 10 mL for the preparation of i-PRF. HbA1c evaluation for all participants was performed at the Central Laboratory of Yenepoya Medical College Hospital, Yenepoya (deemed to be University), to categorize participants into healthy, controlled T2DM, and uncontrolled T2DM groups, and to assess baseline platelet counts at the start of the study.

### 2.3 Separation of A PRF and i-PRF

#### 2.3.1 A-PRF preparation

Venous blood was collected using sterile 10 mL plain red glass vacuum tubes without an anticoagulant and immediately centrifuged using a DUO QUATTRO® PRF centrifuge (Process for PRF, Nice, France; developed by Dr. Joseph Choukroun). The centrifuge is equipped with a fixed-angle rotor (approximately 33° tube angulation). The blood samples were centrifuged at 1,300 rpm for 14 min. Centrifugation was performed using the manufacturer's recommended settings for the DUO QUATTRO PRF centrifuge. Following centrifugation, the fibrin clot formed in the intermediate zone was carefully harvested with sterile forceps, and any accompanying erythrocytes were trimmed away. The harvested clot was then placed in a sterile Eppendorf tube.

#### 2.3.2 i-PRF preparation

Venous blood was collected in anticoagulant-free sterile 13 mL plain purple polypropylene vacuum tubes and and immediately centrifuged using a DUO QUATTRO® PRF centrifuge (Process for PRF, Nice, France; developed by Dr. Joseph Choukroun). The centrifuge is equipped with a fixed-angle rotor (approximately 33° tube angulation) at 700 rpm for 3 min. The yellow supernatant from the upper layer was carefully aspirated with a sterile syringe. Centrifugation was carried out using the Choukroun DUO QUATTRO PRF centrifuge. After preparation, all samples were stored at −80 °C until further analysis, in accordance with the established protocol.

#### 2.3.3 Growth factor analysis

Quantification of growth factors was done to establish the concentration of vascular endothelial growth factor (VEGF), platelet-derived growth factor-BB (PDGF-BB), insulin-like growth factor-1 (IGF-1), transforming growth factor-*β*1 (TGF-*β*1), and transforming growth factor-*β*2 (TGF-*β*2) in both A PRF and i-PRF samples obtained from all study groups. After PRF, the exudates were aliquoted and stored at −80 °C until analysis to prevent protein degradation, with a single thaw before analysis.

The concentration of growth factors was determined using a sandwich enzyme-linked immunosorbent assay (ELISA) kit (Krishgen Biosystems, Mumbai, India; catalog numbers KB1155, KB1100, KBH0103, KB1140, and KBH0067). The kits were used according to the manufacturer's instructions. The sensitivity of the kits was 100 pg/mL for VEGF, 18 pg/mL for PDGF-BB, 0.062 ng/mL for IGF-1, 70.4 pg/mL for TGF-*β*1, and 47 pg/mL for TGF-*β*2. The ranges of the kits were 125–2,000 pg/mL for VEGF, 20–320 pg/mL for PDGF-BB, 0.156–10 ng/mL for IGF-1, 75–2,400 pg/mL for TGF-*β*1, and 50–800 pg/mL for TGF-*β*2.

#### 2.3.4 MTT assay for cell viability

The Methyl Thiazolyl Tetrazolium (MTT) assay was used to determine the effect of A-PRF and i-PRF on cellular proliferation. Following quantification of growth factors, five independent donor samples from each study group with the highest growth factor concentrations were selected for the MTT assay. A-PRF and i-PRF samples from each donor were tested individually and were not pooled. This selection was based on evidence that PRF's biological activity is largely mediated by the release of growth factors that promote cell proliferation, migration, and tissue regeneration. Consequently, PRF samples with higher growth factor concentrations are expected to exhibit greater biological activity and regenerative potential than those with lower concentrations. The objective of this experiment was to functionally evaluate the regenerative potential of the most biologically active PRF preparations rather than to estimate the average biological effect of all PRF samples. Therefore, the MTT assay was performed to functionally validate the most bioactive PRF preparations (17–19).

Murine L929 fibroblast cells were plated in 96-well microtiter plates and maintained in Dulbecco's Modified Eagle's Medium (DMEM) with 10% fetal bovine serum and 1% antibiotic and antimycotic solution at 37 °C in a humidified atmosphere with 5% CO2. After cell attachment, the cells were exposed to A PRF and i-PRF samples and incubated for 24 h. The MTT reagent was then added to each well, and the wells were incubated for 4 h to allow formation of formazan crystals. The crystals were dissolved in dimethyl sulfoxide (DMSO), and the absorbance was measured at 570 nm using a multimode microplate reader (FluoSTAR Omega, BMG Labtech). Cell viability was calculated as a percentage of control cells. Each donor sample was analysed in triplicate (*n* = 3) for both A-PRF and i-PRF preparations.

A PRF and i-PRF were prepared following standard protocols. For *in vitro* assays, PRF samples were added to each cell-containing well at a defined volume of 5 µL. To ensure consistency across groups, the PRF preparations were normalized to equal volumes per well, comparable initial blood volumes used for PRF preparation, and standardized centrifugation conditions. The test samples were applied after initial cell attachment, typically 18 h post-seeding, to evaluate the early cellular response and proliferative potential of PRF preparations in the target cells. This approach allows assessment of PRF's influence on early-stage cellular proliferation. This methodology is consistent with previous studies evaluating biomaterials and platelet concentrates, in which early exposure is critical to mimic the clinical scenario of immediate application to cells/tissues.

### 2.4 Statistical analysis

Data are expressed as mean ± standard deviation (SD). The normality of continuous variables was assessed using the Shapiro–Wilk test. As several outcome variables did not satisfy the assumption of normality, comparisons among the three study groups were performed using the Kruskal–Wallis test, followed by Dunn's multiple comparisons test with Bonferroni adjustment where appropriate.

In addition to group comparisons, multivariable regression analyses were performed to explore the association between glycaemic status, demographic variables, and growth factor release from platelet-rich fibrin preparations. Multiple linear regression models were constructed separately for growth factors derived from advanced platelet-rich fibrin (A PRF) and injectable platelet-rich fibrin (i-PRF), with VEGF, PDGF-BB, and IGF-1 as dependent variables. HbA1c level, age, sex, and diabetes group were included as independent predictors. Multinomial logistic regression analysis was performed using diabetes status (healthy, controlled T2DM, and uncontrolled T2DM) as the outcome variable, with the healthy group as the reference category.

Pearson correlation analysis was performed to evaluate the relationships between HbA1c levels and PRF-derived growth factors, and the correlation structure was visualized using a heatmap. Growth factor distributions were further illustrated using violin plots with embedded boxplots, and statistical significance was assessed using the Kruskal–Wallis test (Table 1) followed by Dunn's multiple comparisons test with Bonferroni adjustment.

**Table 1:** Summary of kruskal–wallis test for comparison of growth factors and platelet count among the groups.

Growth Factor	PRF Type	Comparison	Mean Rank Difference	Adjusted *P-*Value
PDGF-BB	A PRF	Controlled T2DM vs. Uncontrolled T2DM	14.02	0.0832
		Controlled T2DM vs. Healthy Healthy non-diabetic group	−4.28	>0.9999
		Uncontrolled T2DM vs. Healthy Healthy non-diabetic group	−18.3	0.0155
	i-PRF	Controlled T2DM vs. Uncontrolled T2DM	15.93	0.0332
		Controlled T2DM vs. Healthy Healthy non-diabetic group	−7.658	0.6932
		Uncontrolled T2DM vs. Healthy Healthy non-diabetic group	−23.58	0.0008
VEGF	A PRF	Controlled T2DM vs. Uncontrolled T2DM	11.52	0.2113
		Controlled T2DM vs. Healthy Healthy non-diabetic group	0.4867	>0.9999
		Uncontrolled T2DM vs. Healthy Healthy non-diabetic group	−11.04	0.2751
	i-PRF	Controlled T2DM vs. Uncontrolled T2DM	10.81	0.2656
		Controlled T2DM vs. Healthy Healthy non-diabetic group	−1.321	>0.9999
		Uncontrolled T2DM vs. Healthy Healthy non-diabetic group	−12.13	0.1818
IGF-1	A PRF	Controlled T2DM vs. Uncontrolled T2DM	7.74	0.6727
		Controlled T2DM vs. Healthy Healthy non-diabetic group	0.6625	>0.9999
		Uncontrolled T2DM vs. Healthy Healthy non-diabetic group	−7.078	0.8382
	i-PRF	Controlled T2DM vs. Uncontrolled T2DM	14.52	0.0651
		Controlled T2DM vs. Healthy Healthy non-diabetic group	11.36	0.2255
		Uncontrolled T2DM vs. Healthy Healthy non-diabetic group	−3.157	>0.9999
TGF-*β*2	A PRF	Controlled T2DM vs. Uncontrolled T2DM	211.1	0.0744
		Controlled T2DM vs. Healthy Healthy non-diabetic group	−137.5	0.3368
		Uncontrolled T2DM vs. Healthy Healthy non-diabetic group	−348.7	0.0015
	i-PRF	Controlled T2DM vs. Uncontrolled T2DM	247.1	0.0378
		Controlled T2DM vs. Healthy Healthy non-diabetic group	−90.82	0.6228
		Uncontrolled T2DM vs. Healthy Healthy non-diabetic group	−337.9	0.0034
Platelet count		Controlled T2DM vs. Uncontrolled T2DM	5.331	>0.9999
		Controlled T2DM vs. Healthy Healthy non-diabetic group	3	>0.9999
		Uncontrolled T2DM vs. Healthy Healthy non-diabetic group	−2.331	>0.9999

All statistical analyses were performed using R statistical software (version 4.5.2) and GraphPad Prism (version 10). A two-sided *p* value < 0.05 was considered statistically significant.

## 3 Results

### 3.1 Growth factor analysis

#### 3.1.1 PDGF-BB release from A PRF and i-PRF

**Figure 1:**
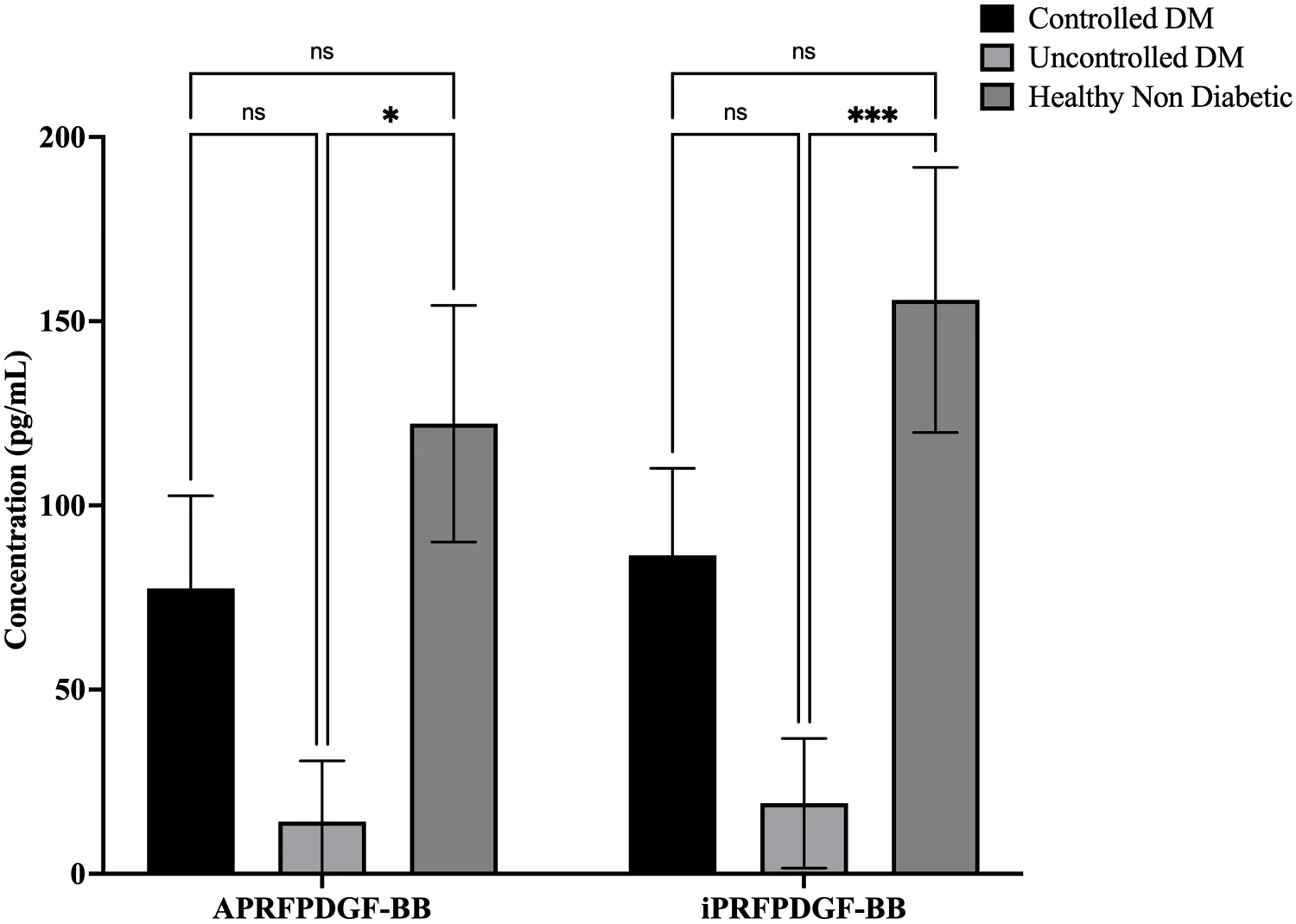
Comparative analysis of PDGF BB in A PRF and i-PRF samples among 3 different groups.

PDGF-BB release was significantly reduced in PRF derived from participants with uncontrolled T2DM (Figure 1). In A-PRF, the healthy non-diabetic group exhibited significantly higher PDGF-BB levels than the uncontrolled T2DM group (*P* = 0.016), whereas no significant difference was observed between the controlled and uncontrolled T2DM groups. In i-PRF, PDGF-BB levels were significantly reduced in the uncontrolled T2DM group compared with the healthy non-diabetic group (*P* = 0.0008) No significant difference was observed between the healthy and controlled T2DM groups. Overall, these findings indicate that poor glycaemic control is associated with reduced PDGF-BB release, whereas PRF obtained from participants with well-controlled T2DM retains PDGF-BB levels comparable to those of healthy individuals.

#### 3.1.2 VEGF release from A PRF and i-PRF

**Figure 2:**
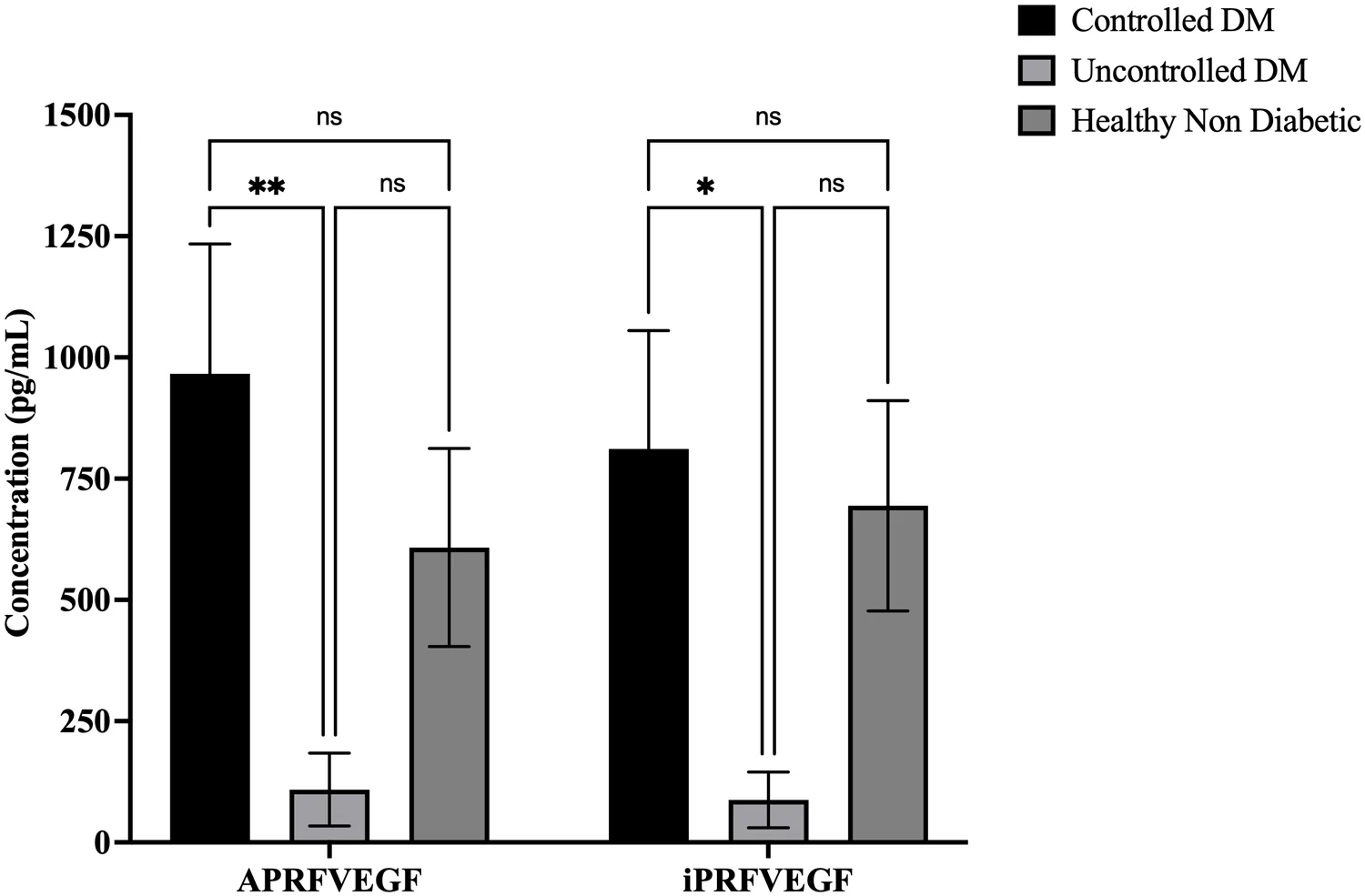
Comparative analysis of VEGF in A PRF and i-PRF samples among 3 different groups.

VEGF release varied according to glycaemic control status (Figure 2). In A-PRF, participants with controlled T2DM exhibited significantly higher VEGF concentrations than those with uncontrolled T2DM (*P* = 0.0096), whereas neither diabetic group differed significantly from the healthy non-diabetic group. A similar pattern was observed in i-PRF, where VEGF concentrations were significantly higher in the controlled T2DM group than in the uncontrolled T2DM group (*P* = 0.0234), with no significant differences between either diabetic group and healthy participants. These findings suggest that effective glycaemic control preserves VEGF release, whereas poor glycaemic control is associated with reduced VEGF levels.

#### 3.1.3 IGF-1 release from A PRF and i-PRF

**Figure 3:**
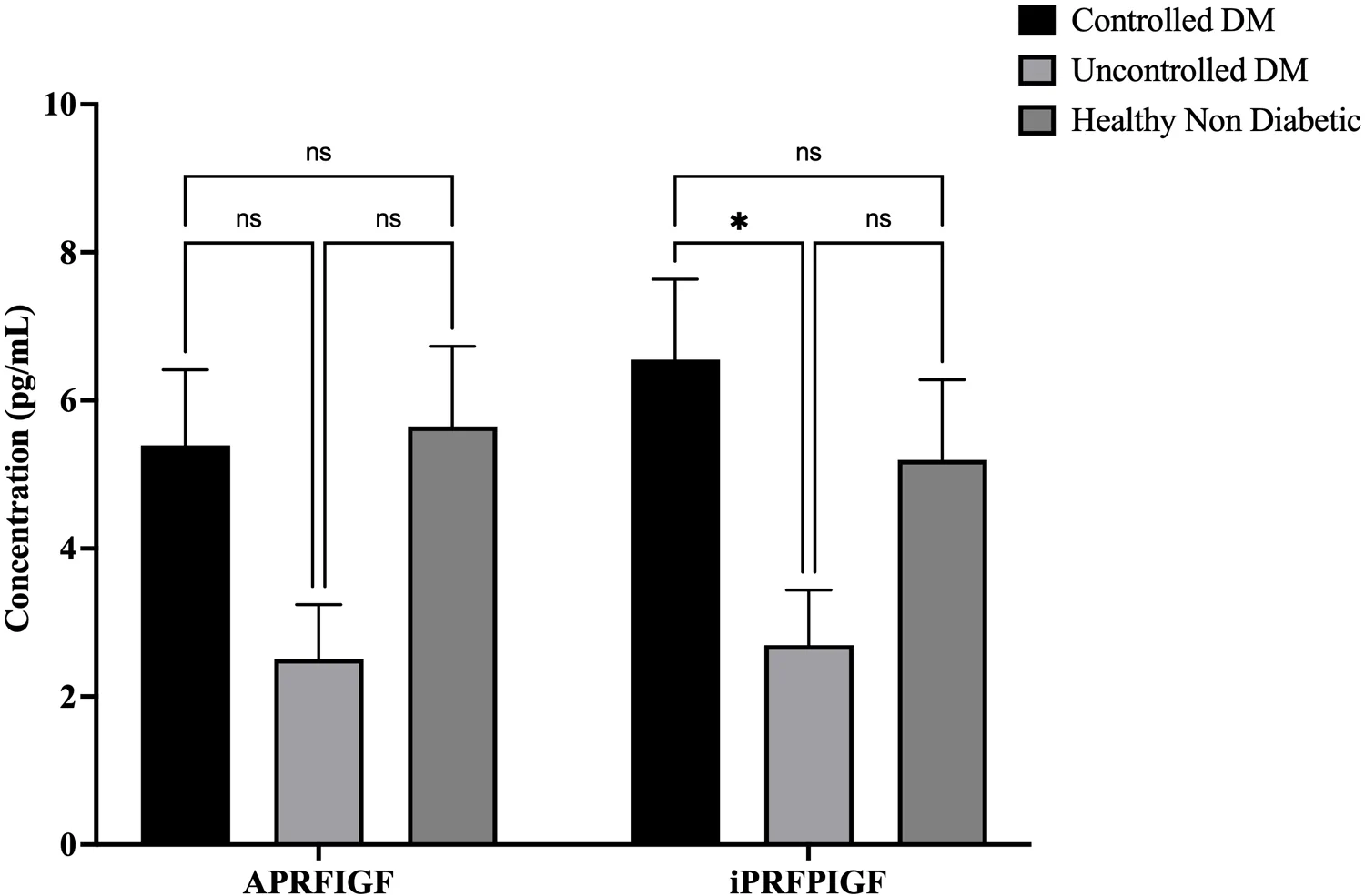
Comparative analysis of IGF-1 in A PRF and i-PRF samples among 3 different groups.

IGF-1 concentrations were broadly comparable among the three study groups (Figure 3). No statistically significant differences were observed in A-PRF preparations. However, in i-PRF, the controlled T2DM group exhibited significantly higher IGF-1 concentrations than the uncontrolled T2DM group (*P* < 0.05), while no significant differences were detected between either diabetic group and the healthy non-diabetic group. These findings suggest that poor glycaemic control may selectively reduce IGF-1 release in i-PRF.

#### 3.1.4 TGF-*β*2 release from A PRF and i-PRF

**Figure 4:**
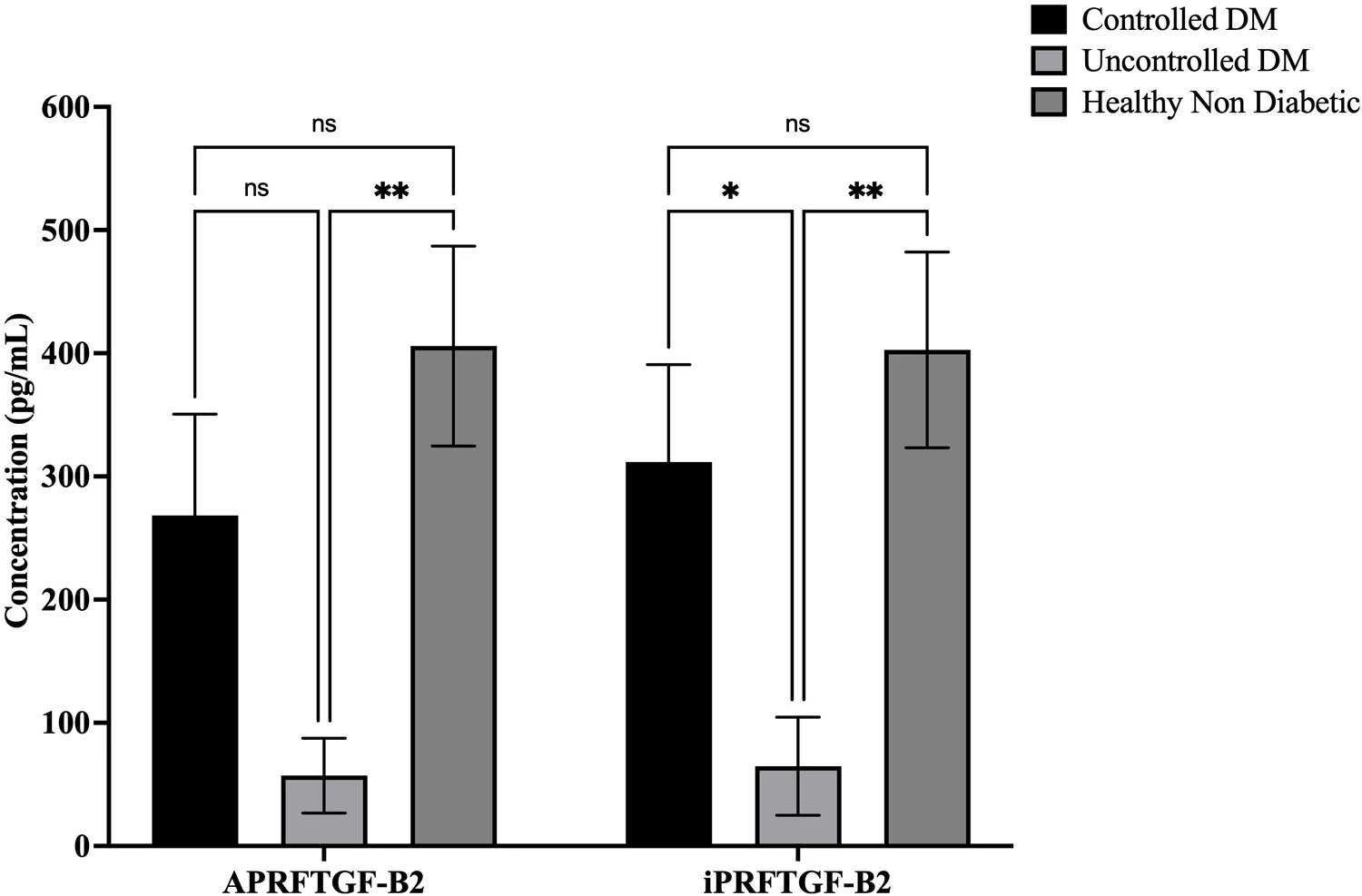
Comparative analysis of TGF *β*2 in A PRF and i-PRF samples among 3 different groups.

TGF-*β*2 release was also reduced in PRF derived from participants with uncontrolled T2DM (Figure 4). TGF-*β*2 release from A PRF showed no significant difference between the controlled T2DM and uncontrolled T2DM groups (*P* > 0.05), although the controlled group demonstrated relatively higher levels. The healthy non-diabetic group showed significantly higher levels compared with the uncontrolled T2DM group (*p* = 0.002). A similar pattern was observed in i-PRF, with the uncontrolled T2DM group exhibiting significantly lower TGF-*β*2 levels than the Healthy non-diabetic group (*p* = 0.0034).

### 3.2 Comparison of platelet count among three groups

**Figure 5:**
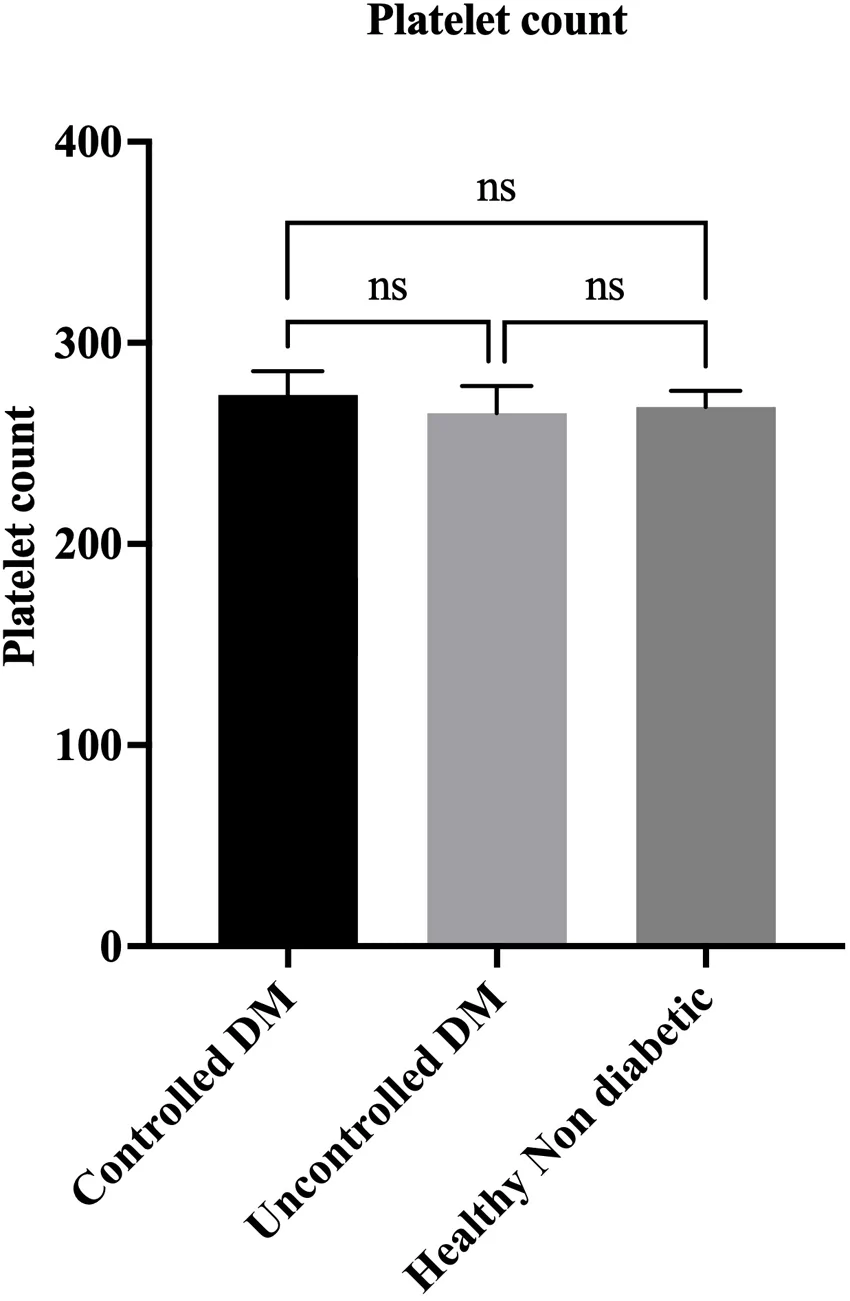
Comparison of the platelet counts among 3 groups.

Platelet counts were comparable among the controlled T2DM, uncontrolled T2DM, and healthy non-diabetic groups, with no statistically significant differences observed between any pair of groups (Figure 5).

### 3.3 Effect of A PRF and i-PRF on L929 cell viability

**Figure 6:**
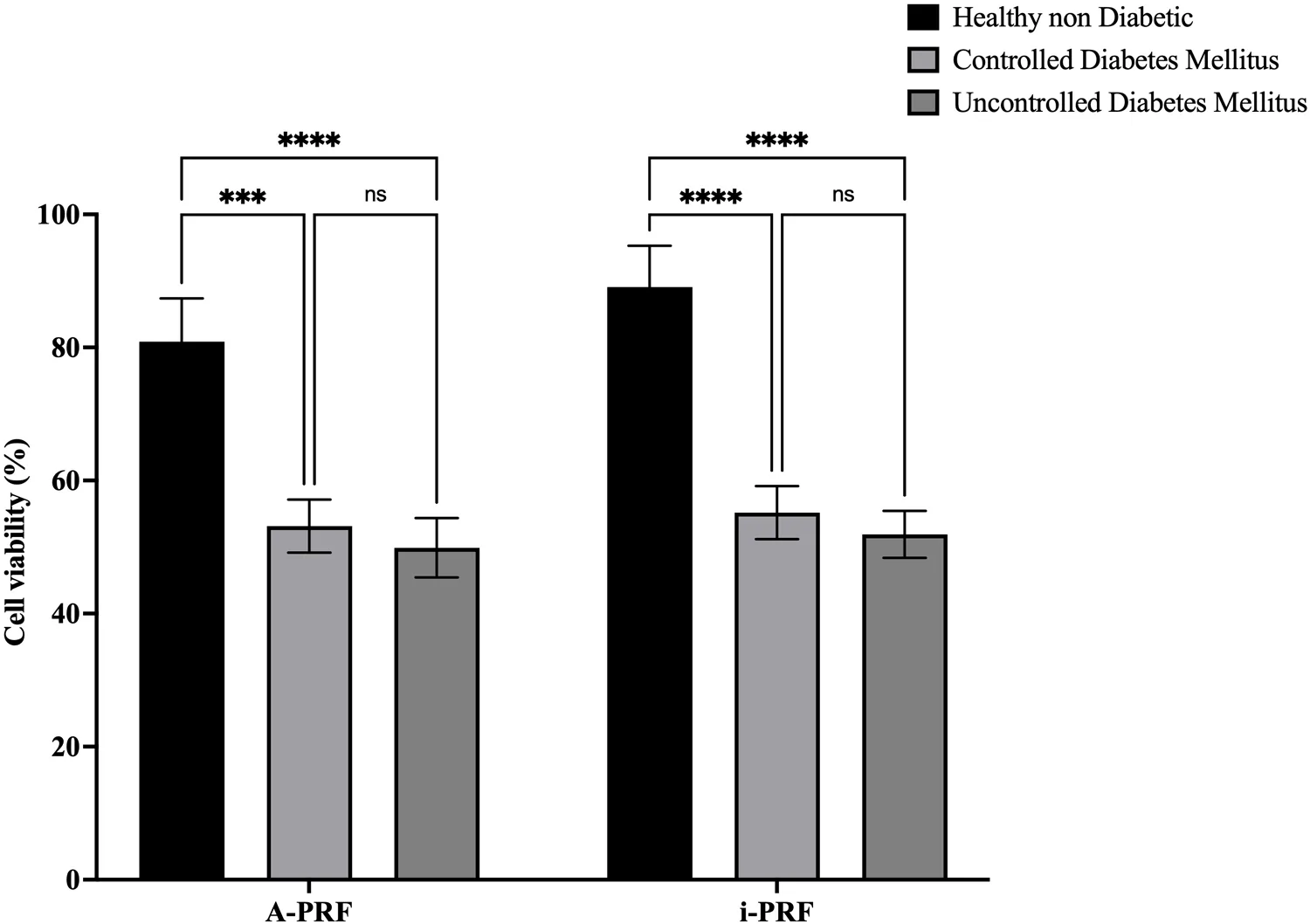
Effect of A PRF and i-PRF on the viability of L929 cells.

As illustrated in Figure 6, both A PRF and i-PRF samples from the healthy non-diabetic group demonstrated significantly higher L929 cell viability compared with samples from the controlled T2DM and uncontrolled T2DM [*p* = 0.0013 (mean difference = 27.73) and 0.0004 (mean difference =30.99) respectively for A-PRF and *p* < 0.0001 for iPRF (mean difference = 33.92 and 37.18 respectively)], indicating enhanced proliferative potential in the healthy group.

### 3.4 Correlation analysis of platelet count and growth factors

As summarized in Tables 2–4, no significant correlations were observed between platelet counts and growth factor levels (PDGF-BB, VEGF, IGF-1, and TGF-*β*2) in either A PRF or i-PRF samples across the three groups (*p* > 0.05).

**Table 2:** Correlation between platelet count and growth factor levels in A PRF and i-PRF samples from the Healthy non-diabetic group group.

Pearson r	Platelet count vs. PDGF BB A PRF	Platelet count vs. IGF 1 A PRF	Platelet count vs. VEGF A PRF	Platelet count vs. TGF β2 A PRF	Platelet count vs. PDGF BB i-PRF	Platelet count vs. IGF 1 i-PRF	Platelet count vs. VEGF i-PRF	Platelet count vs. TGF β2 i-PRF
r	0.3245	0.307	0.3191	0.2136	0.334	0.2919	0.3144	0.02167
95% confidence interval	−0.07187 to 0.6324	−0.09116 to 0.6206	−0.07787 to 0.6287	−0.1982 to 0.5614	−0.07040 to 0.6441	−0.1076 to 0.6103	−0.08306 to 0.6256	−0.3688 to 0.4056
R squared	0.1053	0.09427	0.1018	0.04564	0.1116	0.08523	0.09885	0.0004694
*P* value *P* (two-tailed)	0.1058	0.1271	0.1121	0.3052	0.1027	0.1479	0.1178	0.9163
*P* value summary	ns	ns	ns	ns	ns	ns	ns	ns
Significant? (alpha = 0.05)	no	no	no	no	no	no	no	no
Number of XY Pairs	26	26	26	25	25	26	26	26

**Table 3:** Correlation between platelet count and growth factor levels in A PRF and i-PRF samples from the controlled diabetic group.

Pearson r	Platelet count vs. PDGF BB A PRF	Platelet count vs. IGF 1 A PRF	Platelet count vs. VEGF A PRF	Platelet count vs. TGF β2 A PRF	Platelet count vs. PDGF BB i-PRF	Platelet count vs. IGF1 i-PRF	Platelet count vs. VEGF i-PRF	Platelet count vs. TGF β2 i-PRF
r	0.2361	0.09948	0.2626	0.0592	0.1626	0.1886	0.1477	−0.2466
95% confidence interval	−0.1427 to 0.5546	−0.2771 to 0.4496	−0.1149 to 0.5739	−0.3359 to 0.4365	−0.2241 to 0.5050	−0.2062 to 0.5306	−0.2385 to 0.4936	−0.5729 to 0.1472
R squared	0.05575	0.009896	0.06897	0.003505	0.02643	0.03557	0.02182	0.06081
*P* value *P* (two-tailed)	0.2175	0.6077	0.1687	0.7739	0.4085	0.3461	0.4532	0.215
*P* value summary	ns	ns	ns	ns	ns	ns	ns	ns
Significant? (alpha = 0.05)	no	no	no	no	no	no	no	no
Number of XY Pairs	29	29	29	26	28	27	28	27

**Table 4:** Correlation between platelet count and growth factor levels in A PRF and i-PRF samples from the uncontrolled diabetic group.

Pearson r	Platelet count vs. PDGF BB A PRF	Platelet count vs. IGF- A PRF	Platelet count vs. VEGF A PRF	Platelet count vs. TGF β2 A PRF	Platelet count vs. PDGF BB i-PRF	Platelet count vs. IGF- i-PRF	Platelet count vs. VEGF i-PRF	Platelet count vs. TGF β2 i-PRF
r	0.07848	−0.2081	0.1193	0.06972	−0.1727	−0.1778	0.06049	−0.2403
95% confidence interval	−0.3108 to 0.4452	−0.5450 to 0.1867	−0.2731 to 0.4777	−0.3187 to 0.4381	−0.5187 to 0.2219	−0.5225 to 0.2168	−0.3270 to 0.4306	−0.5803 to 0.1711
R squared	0.006159	0.0433	0.01423	0.004861	0.02983	0.03162	0.0215	0.05774
*P* value *P* (two-tailed)	0.6972	0.2976	0.5534	0.7297	0.389	0.3749	0.4655	0.2473
*P* value summary	ns	ns	ns	ns	ns	ns	ns	ns
Significant? (alpha = 0.05)	no	no	no	no	no	no	no	no
Number of XY Pairs	27	27	27	27	27	27	27	25

### 3.5 Correlation between HbA1c and PRF-derived growth factors

**Figure 7:**
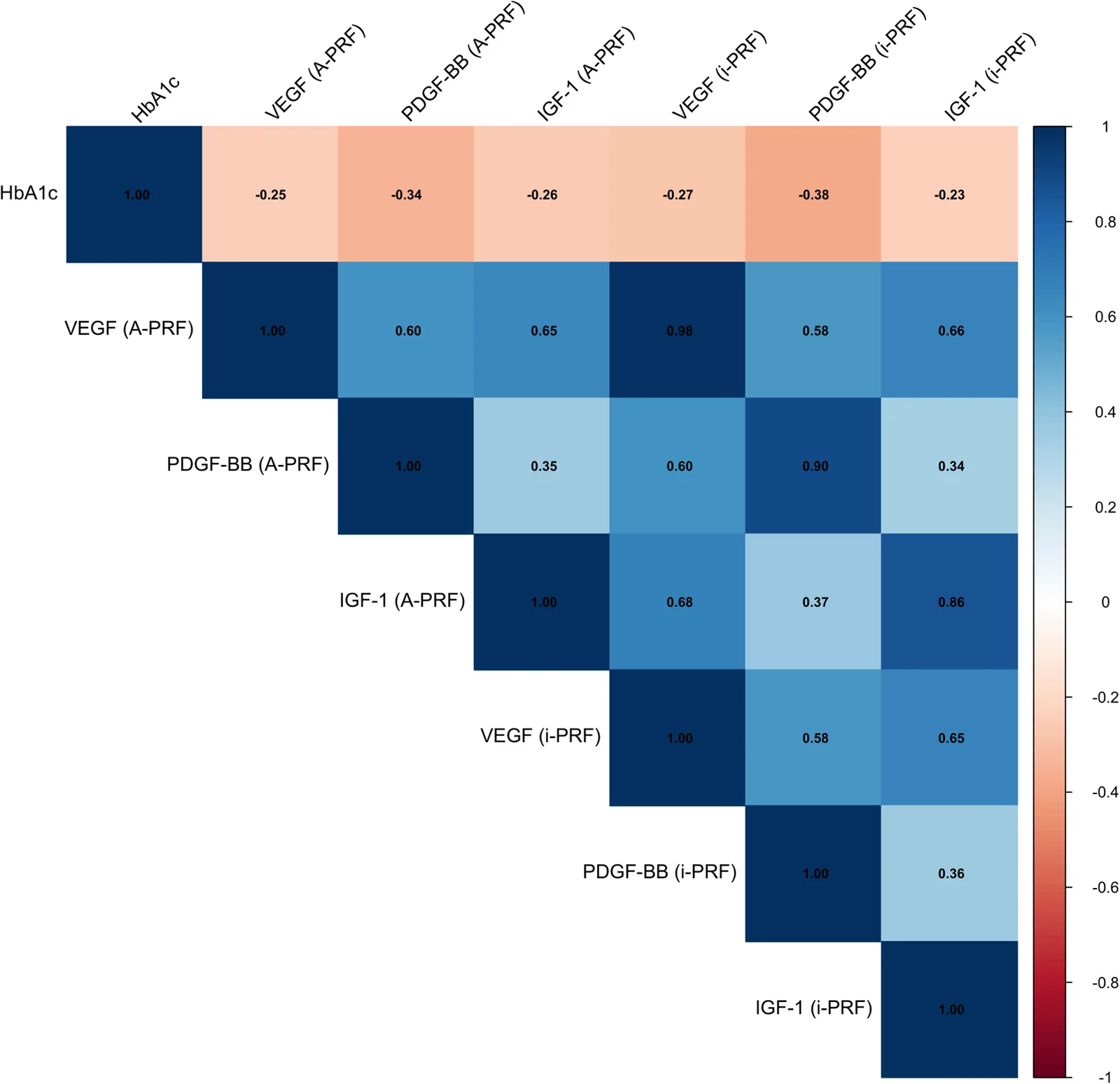
Correlation heatmap of HbA1c and PRF-derived growth factors. Heatmap illustrating pairwise Pearson correlation coefficients between HbA1c and growth factors released from advanced platelet-rich fibrin (A PRF) and injectable platelet-rich fibrin (i-PRF). Variables included HbA1c, VEGF, PDGF-BB, and IGF-1 measured in both PRF preparations. Color intensity represents the strength and direction of the correlation, ranging from negative correlations (red) to positive correlations (blue). Strong positive correlations were observed among growth factors within PRF preparations, whereas HbA1c showed weak or negative correlations with most growth factor levels. The upper triangular matrix is shown to avoid redundancy and improve visualization clarity.

Correlation analysis was further performed to evaluate the relationship between glycaemic control and the release of PRF-derived growth factors. The correlation heatmap (Figure 7) illustrates the pairwise Pearson correlation coefficients between HbA1c levels and growth factors derived from both A PRF and i-PRF preparations. Strong positive correlations were observed among growth factors within the same PRF preparation, particularly among VEGF, PDGF-BB, and IGF-1, indicating coordinated release patterns from the fibrin matrix. In contrast, HbA1c showed weak or negative correlations with most growth factor levels, suggesting that glycaemic status alone was not a strong determinant of PRF-derived growth factor concentrations in this cohort.

### 3.6 Relationship between HbA1c and growth factor release

**Figure 8:**
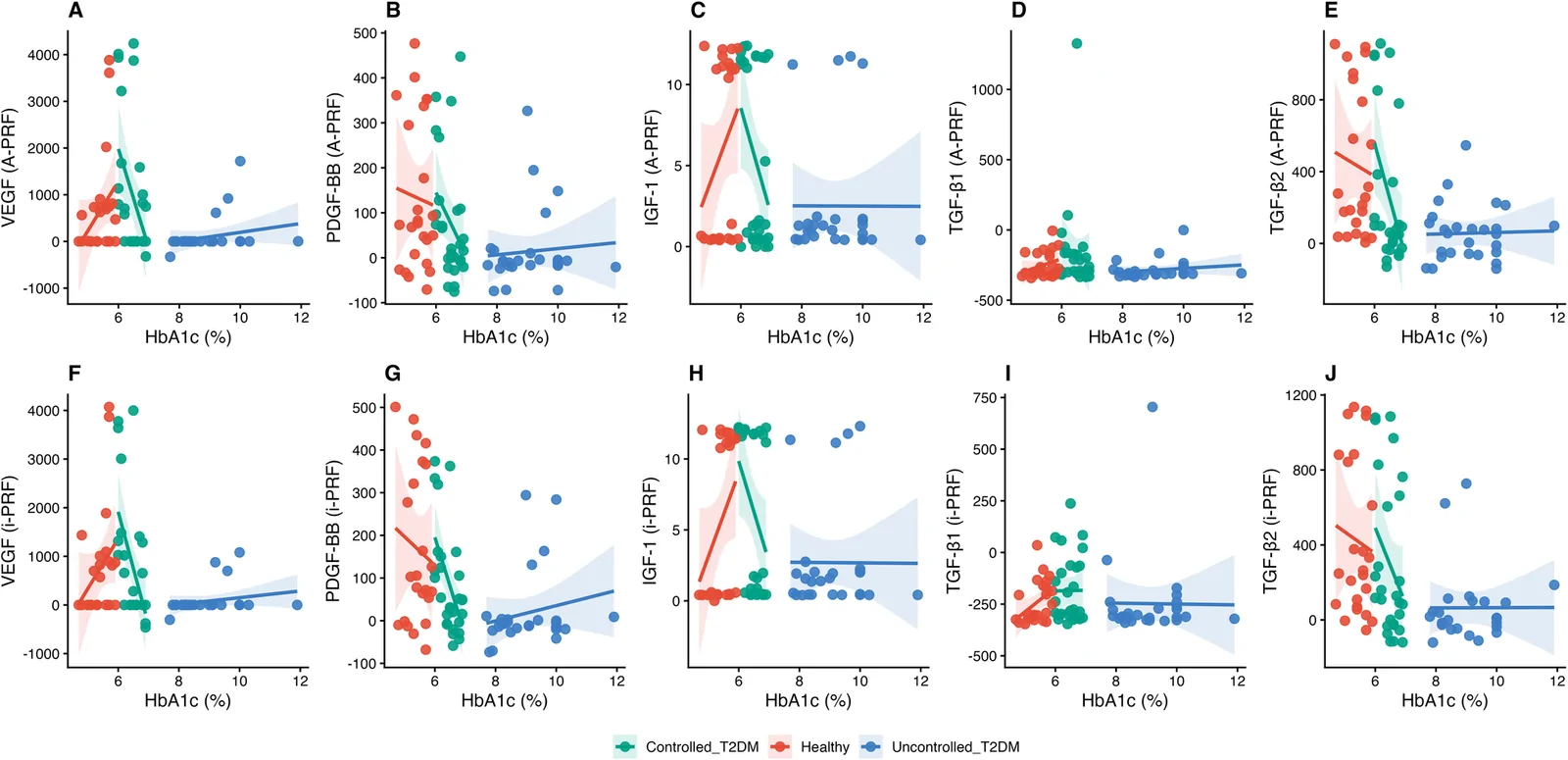
Relationship between HbA1c levels and growth factor release from platelet-rich fibrin preparations. Scatter plots illustrate the association between HbA1c and the concentrations of VEGF, PDGF-BB, and IGF-1 released from A PRF (panels **A–C**) and i-PRF (panels **D–F**). Points represent individual samples colored by study group (healthy, controlled T2DM, uncontrolled T2DM). Solid lines represent linear regression fits with shaded 95% confidence intervals. Panels **A–C** show VEGF, PDGF-BB, and IGF-1 levels in A PRF, while panels **D–F** show the corresponding growth factors in i-PRF. The plots illustrate the distribution of growth factor release across varying levels of glycaemic control.

Scatter plot analysis was performed to evaluate the association between HbA1c levels and the release of key growth factors from A PRF and i-PRF preparations (Figure 8). Across both PRF types, the regression lines demonstrated relatively weak relationships between HbA1c and the concentrations of VEGF, PDGF-BB, and IGF-1. Although some variability in growth factor release was observed across the range of HbA1c values, no clear linear trend was evident, indicating that increasing glycaemic burden was not strongly associated with proportional changes in growth factor concentrations. Samples from controlled T2DM participants generally overlapped with those from healthy participants, whereas samples from uncontrolled T2DM participants showed greater variability and a tendency toward lower growth factor levels, particularly for VEGF. Correlation and regression analyses demonstrated that HbA1c was not independently associated with most PRF-derived growth factors. Although VEGF release exhibited differences between glycaemic groups in selected analyses, increasing HbA1c values alone did not demonstrate a consistent linear relationship with growth factor concentrations, indicating that categorical glycaemic status may better explain biological variation than HbA1c as a continuous measure.

### 3.7 Distribution of growth factors among groups

**Figure 9:**
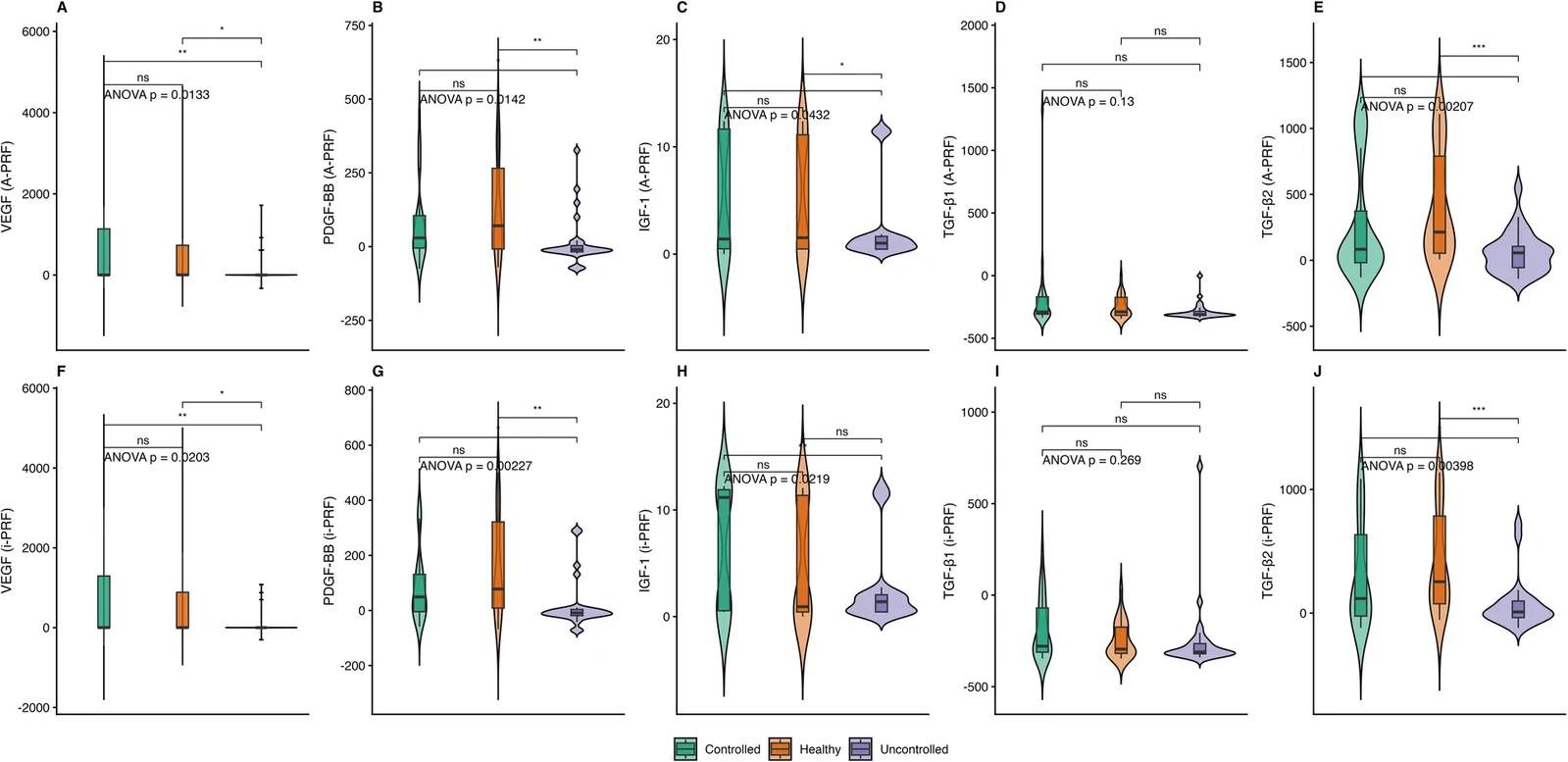
Comparison of growth factor release among study groups. Violin plots with embedded boxplots illustrate the distribution of VEGF, PDGF-BB, IGF-1, TGF-*β*1, and TGF-*β*2 released from advanced platelet-rich fibrin (A PRF; panels **A–E**) and injectable platelet-rich fibrin (i-PRF; panels **F–J**) across healthy participants, controlled T2DM, and uncontrolled T2DM groups. Differences between groups were evaluated using one-way ANOVA followed by Bonferroni-adjusted pairwise comparisons. Significant pairwise differences are indicated on the plots.

The distribution of growth factor concentrations across study groups was further visualized using violin plots with embedded boxplots (Figure 9). For A PRF preparations (panels A–E), VEGF levels were significantly higher in the controlled T2DM group compared with the uncontrolled T2DM group, while PDGF-BB and TGF-*β*2 showed significantly higher levels in healthy participants relative to uncontrolled T2DM participants. IGF-1 and TGF-*β*1 did not demonstrate statistically significant differences among groups. Similar patterns were observed for i-PRF preparations (panels F–J), in which VEGF and TGF-*β*2 levels were significantly reduced in the uncontrolled T2DM group compared with both healthy and controlled T2DM participants. Overall, these distributions indicate that PRF derived from participants with controlled T2DM generally exhibited growth factor profiles comparable to those of healthy non-diabetic participants, whereas uncontrolled T2DM was associated with reduced Growth factor release.

### 3.8 Multivariate regression analyses

Multiple linear regression analyses were performed to evaluate the association between glycaemic status, demographic variables, and the levels of growth factors released from A PRF and i-PRF preparations. Across both PRF types, HbA1c levels were not significantly associated with any of the evaluated growth factors, including VEGF, PDGF-BB, IGF-1, TGF-*β*1, or TGF-*β*2. Similarly, Diabetes status did not show a statistically significant independent association with most growth factor levels after adjusting for age and sex (Tables 5, 6).

**Table 5:** Linear regression models for A PRF growth factors

Predictor	VEGF (A PRF) β (SE)	p	PDGF (A PRF) β (SE)	p	IGF (A PRF) β (SE)	p
HbA1c	60.45 (184.94)	0.745	−7.48 (23.52)	0.751	0.16 (0.85)	0.855
Age	−7.26 (13.55)	0.594	−2.79 (1.72)	0.11	0.07 (0.06)	0.264
Male sex	**−690.67** (**245.55)**	**0**.**006**	−6.06 (31.22)	0.847	**−3.15** (**1.13)**	**0**.**006**
Healthy	−460.67 (371.81)	0.219	20.03 (47.28)	0.673	0.74 (1.72)	0.668
Uncontrolled T2DM	−964.49 (557.86)	0.088	−44.14 (70.93)	0.536	−3.03 (2.58)	0.244

Model statistics:			
Outcome	R^2^	Adj R^2^	Model p
VEGF A PRF	0.192	0.137	0.0066
PDGF A PRF	0.15	0.092	0.0318
IGF A PRF	0.2	0.146	0.0048

Bold values indicate statistically significant results (*p* < 0.05), including regression coefficients and corresponding *p*-values.

**Table 6:** Linear regression models for i-PRF growth factors

Predictor	VEGF (i-PRF) β (SE)	p	PDGF (i-PRF) β (SE)	p	IGF (i-PRF) β (SE)	p
HbA1c	15.49 (173.16)	0.929	−6.11 (24.17)	0.801	0.30 (0.84)	0.728
Age	−1.26 (12.69)	0.921	−2.92 (1.77)	0.102	0.02 (0.06)	0.788
Male sex	**−652.88** (**231.71)**	**0**.**006**	−23.72 (32.21)	0.464	**−4.20** (**1.14)**	**0**.**0004**
Healthy	−209.87 (349.89)	0.55	42.41 (48.74)	0.387	−1.52 (1.71)	0.375
Uncontrolled T2DM	−726.51 (523.79)	0.17	−50.53 (73.03)	0.491	−4.33 (2.57)	0.096

Model statistics:			
Outcome	R^2^	Adj R^2^	Model p
VEGF i-PRF	0.192	0.136	0.0073
PDGF i-PRF	0.202	0.146	0.0055
IGF i-PRF	0.251	0.199	0.0007

To further explore whether PRF-derived growth factors were associated with diabetes status, a multinomial logistic regression model was fitted with the healthy group as the reference category. In this model, higher VEGF levels in A PRF were significantly associated with increased odds of belonging to the controlled T2DM group relative to healthy participants. Conversely, lower VEGF levels in i-PRF were significantly associated with increased odds of belonging to the uncontrolled T2DM group. These results suggest that differential VEGF release from the two PRF preparations may reflect variations in glycaemic control (Table 7).

Bold values indicate statistically significant results (*p* < 0.05), including regression coefficients and corresponding *p*-values.

**Table 7:** Multinomial logistic regression predicting diabetes status. Healthy participants were used as the reference group.

Predictor	Controlled T2DM β	p	Uncontrolled T2DM β	p
VEGF A PRF	**5**.**12**	**0**.**036**	5.55	0.054
VEGF i-PRF	−3.71	0.118	**−5**.**93**	**0**.**035**
PDGF A PRF	−0.51	0.652	0.6	0.566
PDGF i-PRF	−1.04	0.229	−1.57	0.111
IGF A PRF	−4.82	0.073	−4.01	0.131
IGF i-PRF	5.28	0.058	4.12	0.136
Age	**0**.**21**	**0**.**00027**	**0**.**19**	**0**.**00044**
Male sex	**3**.**25**	**0**.**004**	**2**.**32**	**0**.**032**

Demographic variables also demonstrated strong directional associations with diabetes status. Increasing age was positively associated with both controlled and uncontrolled T2 T2DM groups, indicating that older participants had higher odds of diabetes compared with healthy participants. Likewise, male sex was positively associated with diabetes status, with males showing significantly greater odds of being classified in both controlled and uncontrolled T2 T2DM groups relative to females. Collectively, these findings indicate that while most PRF-derived growth factors were not directly associated with glycaemic status, VEGF profiles and demographic factors, particularly advancing age and male sex, show meaningful associations with diabetes classification in this cohort.

Bold values indicate statistically significant results (*p* < 0.05), including regression coefficients and corresponding *p*-values.

However, sex demonstrated a consistent and significant effect on several growth factor outcomes. Male participants exhibited significantly lower levels of VEGF and IGF-1 in both A PRF and i-PRF preparations compared with females. In addition, males showed significantly lower TGF-*β*1 levels in both A PRF and i-PRF. These findings suggest that sex-related biological differences may influence the release of certain PRF-derived growth factors, independent of glycaemic control or diabetes status. Age was not significantly associated with any of the growth factor outcomes in the adjusted models.

## 4 Discussion

Periodontitis is a chronic inflammatory disease primarily caused by dental plaque, whose progression is influenced by systemic conditions such as T2DM, which is associated with impaired wound healing and compromised periodontal treatment outcomes (20). Platelet-rich fibrin (PRF) has emerged as a promising autologous biomaterial for periodontal regeneration due to its sustained release of growth factors that promote tissue repair (9). However, limited information is available regarding the biological properties of PRF derived from individuals with varying levels of glycaemic control. Previous studies suggest that advanced platelet-rich fibrin (A PRF) can enhance natural healing, accelerate tissue regeneration, and improve postoperative comfort (14). The limited literature evaluating the use of advanced platelet concentrates in diabetic participants prompted the design of the current laboratory-based analysis of human-derived PRF to explore its quantitative and qualitative characteristics in diabetic participants compared with Healthy non-diabetic participants.

In the present study, platelet quantification and growth factor analysis of autologous A PRF and i-PRF were evaluated and compared among the controlled T2DM, uncontrolled T2DM, and Healthy non-diabetic groups. Platelet counts did not differ substantially among the groups, indicating that chronic hyperglycaemia does not significantly influence mean platelet counts in diabetic participants. These findings are consistent with prior investigations (21–23), which also observed no variation in platelet counts between T2DM and Healthy non-diabetic group participants. Conversely, other studies (24, 25) have reported elevated platelet counts in diabetic participants compared with healthy non-diabetic groups. Variations in platelet count in diabetes may be influenced by factors such as platelet lifespan, production rate, and turnover (24). Importantly, the biological behavior of platelets in diabetes is determined predominantly by qualitative functional alterations rather than absolute platelet count. Persistent hyperglycaemia has been reported to alter platelet behavior through several mechanisms, including protein glycation, oxidative stress, and intracellular signaling abnormalities (26). These changes may modify platelet activation and, consequently, influence the release of growth factors from PRF (42).

The quantification of Growth factors, namely PDGF-BB, IGF, VEGF, and TGF-*β*2 released from A PRF and i-PRF samples, was quantified using ELISA on all three groups of the study. The results show that VEGF (from both A PRF and i-PRF), IGF (from i-PRF), and TGF-*β*2 (from i-PRF) were significantly higher in the controlled T2DM than in the uncontrolled T2DM. There were no significant differences between the controlled T2DM and the Healthy non-diabetic group. On the other hand, PDGF-BB (from both A PRF and i-PRF) and TGF-*β*2 (from both A PRF and i-PRF) were significantly higher in the Healthy non-diabetic group than in the uncontrolled T2DM group. There were no significant differences between the Healthy non-diabetic group and the controlled T2DM groups. These findings suggest that PRF derived from individuals with well-controlled T2DM exhibited laboratory-based biological characteristics, including growth factor release profiles, comparable to those observed in healthy individuals. The results can be attributed to the differences in the structural characteristics of the fibrin matrix. PRF functions through a three-dimensional polymerized fibrin network that entraps platelets and leukocytes, allowing gradual release of bioactive molecules during clot maturation. Variations in the architecture of this fibrin matrix may influence both the amount and the kinetics of growth factor release (18, 27). However, because HbA1c was not independently associated with most growth factor outcomes in the adjusted analyses, these observations should be interpreted as group-level differences rather than evidence that glycaemic control alone determines PRF biological properties.

A recent study reported comparable PDGF-BB levels among healthy participants, T2DM participants, participants with periodontitis, and those with both T2DM and periodontitis, whereas TGF-*β*1 levels were highest in healthy participants (28). These observations indicate that platelet activation may not be substantially impaired in participants with well-controlled T2DM. The consistent finding that A PRF and i-PRF obtained from controlled diabetic participants exhibit growth factor release patterns comparable to those of healthy non-diabetic group participants, whereas uncontrolled T2DM shows significantly reduced levels, suggests that predictable regenerative outcomes may be achievable in controlled T2DM through the use of platelet concentrates. However, these findings should be interpreted cautiously, as the present study did not evaluate clinical regenerative outcomes.

An additional objective of the present study was to determine whether growth factor release from PRF preparations was independently associated with glycaemic status after accounting for demographic factors. Multivariable regression analysis showed that HbA1c levels were not significantly associated with most growth factor outcomes, suggesting that systemic glycaemic burden alone may not directly determine the amount of growth factors released from platelet concentrates. Interestingly, VEGF derived from A PRF showed a positive association with controlled T2DM status, whereas VEGF derived from i-PRF was negatively associated with uncontrolled T2DM. VEGF plays a central role in angiogenesis and tissue repair, and differences in VEGF release between PRF preparations may reflect variations in fibrin architecture and platelet activation dynamics under different metabolic conditions. Furthermore, demographic variables such as age and sex demonstrated strong associations with diabetes classification, indicating that patient-specific biological characteristics may influence both systemic metabolic status and PRF-derived regenerative potential. These findings highlight the complex interaction between host metabolic status, platelet biology, and fibrin matrix structure in determining the biological activity of PRF preparations.

We note here that the unadjusted group comparisons evaluated differences among predefined clinical categories (healthy, controlled T2DM, and uncontrolled T2DM), whereas the multivariable regression models assessed the independent contribution of HbA1c after adjustment for age, sex, and diabetes status. Although significant differences in VEGF and TGF-*β*2 release were observed among the healthy, controlled T2DM, and uncontrolled T2DM groups, HbA1c was not consistently identified as an independent predictor in the multivariable regression analyses. Therefore, HbA1c, treated as a continuous predictor, is not independently associated with most growth-factor outcomes. This finding suggests that categorizing participants by glycaemic status may better distinguish differences in PRF biological properties than HbA1c alone does in this cohort.

The biological mechanisms underlying the observed differences remain unclear because they were not directly evaluated in this study. Nevertheless, previous reports suggest that improved glycaemic control may preserve platelet function and fibrin matrix integrity by reducing oxidative stress and protein glycation, thereby potentially influencing growth factor release from PRF (29, 30).

The present study also evaluated the correlation between platelet counts and growth factor release across all groups. No significant correlation was observed between platelet count and the release of PDGF-BB, IGF, VEGF, or TGF-*β*2. These findings suggest that platelet activation and growth factor release may not be directly dependent on platelet count alone in participants with T2DM. Irrespective of glycaemic status, the observed platelet counts and growth factor profiles indicate that the relationship between T2DM and platelet function is complex and influenced by multiple biological factors.

In addition, cell viability analysis revealed that the healthy non-diabetic group exhibited significantly higher cell viability than the control T2DM group in the L929 fibroblast cell line. L929 mouse fibroblasts have been widely employed in cell proliferation studies, including investigations involving turmeric-derived nanoparticles (31) and *Clinacanthus nutans* extracts at varying concentrations (32). The L929 fibroblast line was chosen as an initial *in-vitro* screening model due to its robust growth characteristics, ease of culture, and reproducible proliferative responses, making it appropriate for preliminary assessment of the cytocompatibility and bioactivity of biomaterial-derived preparations such as PRF. However, as a murine continuous cell line, it does not fully replicate the tissue-specific phenotype, extracellular matrix interactions, and regenerative behavior of human periodontal ligament fibroblasts; thus, the findings should be interpreted as preliminary and require validation in human PDL cells before direct clinical extrapolation (33–35).

Previous studies (36, 37) have also reported higher cell viability under low-glucose conditions than under high-glucose conditions in human periodontal ligament fibroblasts and human gingival fibroblasts. This may reflect an adaptive response in diabetic participants, wherein altered cellular mechanisms promote survival under chronic hyperglycaemic stress.

Despite these observations, the release of growth factors may not be sufficient to effectively stimulate cell proliferation, potentially limiting the regenerative capacity of PRF preparations. Furthermore, the storage conditions and duration of PRF samples may have influenced their biological activity, as prolonged storage can degrade essential cellular components and growth factors, reducing cell proliferation and regenerative effectiveness.

Under normal glycaemic conditions, immune cell function is preserved, facilitating effective chemotaxis and phagocytosis (38). Conversely, hyperglycaemia in both uncontrolled and controlled T2DM can lead to increased oxidative stress, accumulation of advanced glycation end-products (AGEs), and impaired intracellular signaling, all of which negatively affect cell viability (39). Oxidative stress and AGEs have been widely reported as central contributors to the pathogenesis and progression of diabetes and its associated complications (40, 41).

The findings of this study suggest that glycaemic control may influence the biological properties of A-PRF and i-PRF. However, because the study was limited to laboratory analyses of PRF preparations obtained from a cohort of participants, together with an *in vitro* cell viability assay, the observed differences in growth factor release and fibroblast viability should not be interpreted as evidence of improved clinical periodontal regeneration. Future well-designed clinical trials with long-term follow-up are needed to determine whether these biological findings translate into improved periodontal treatment outcomes in patients with diabetes. Future investigations should also consider matching or adjusting for sex and accounting for diabetes duration and medication use to minimize potential confounding.

### 4.1 Limitations

This study has certain limitations that should be considered while interpreting the findings. The present study was based on laboratory analyses of PRF preparations of study participants and therefore cannot determine whether the observed biological differences translate into periodontal wound-healing or regenerative outcomes in clinical practice. Cell viability was assessed using the L929 murine fibroblast cell line, which is widely used for preliminary cytocompatibility testing but may not completely reflect the biological behavior of human periodontal ligament fibroblasts. Although the study groups were comparable in size, participants were not matched for sex, and our regression analysis suggested that sex may influence the release of certain PRF-derived growth factors. In addition, factors such as the duration of diabetes and variations in antidiabetic medication use were not evaluated and may have influenced the biological characteristics of PRF. Finally, while standardized protocols were followed, PRF handling and storage could have affected growth factor stability and bioactivity. Since the present study was limited to laboratory-based analyses, the findings should be interpreted as biological evidence and cannot be directly extrapolated to clinical periodontal regenerative outcomes.

## 5 Conclusions

The present study demonstrated that A-PRF and i-PRF derived from participants with well-controlled T2DM exhibited biological properties comparable to those of healthy individuals, whereas uncontrolled diabetes was associated with reduced growth factor release. These findings suggest that achieving good glycaemic control is essential, as it provides biological evidence supporting the potential use of PRF in patients with well-controlled T2DM. However, HbA1c was not independently associated with most growth factor outcomes in the adjusted analyses. The present study's observations are based on laboratory analysis of PRF with an *in vitro* cell viability assay; clinical confirmation through well-designed prospective trials remains essential before these findings can be translated into routine periodontal practice.

## Data Availability

The original contributions presented in the study are included in the article/Supplementary Material, further inquiries can be directed to the corresponding author.
